# Incidents of Animal Life

**Published:** 1887-08-27

**Authors:** F. O. Morris

**Affiliations:** Rector of Nunburnholme, Yorkshire; author of "A History of British Birds," etc


### INCIDENTS OF ANIMAL LIFE.

COLLATED BY THE REV. F. O. MORRIS, B.A.,

Rector of Nunburnholme, Yorkshire; author of "A History of British Birds," etc.

"He prayeth best, who loveth best
All things both great and small;
For the dear God who loveth us,
He made and loveth all."—COLERIDGE.

### ABOUT BIRDS.

THE following touching little incident may interest you as a lover of "things both great and small." In the summer of 1884 my wife had a pair of peroquets given to her. One of them was quite tame, and would rest on her hand, and feed even while she walked about the room; the other bird was wild, and refused to become friendly. But one day the unfriendly bird grew as tame as its mate. The next morning it was found dead in its cage! Soon afterwards its loss was replaced, and the new-comer was also shy, and continued to be so until yesterday, when my wife was charmed to have both birds running about her table, and even pecking at the food on her plate. Alas! most sad to relate, both birds are dead this morning. I cannot discover the cause of their death.

---

We have at this rectory this winter a dear little friend—a robin redbreast—as a daily visitor at breakfast and luncheon, as regularly as the meal comes on. He does everything but speak to us, we sometimes think. He has sat upon my wife's cap and my own arm, though not often nor for long. He pounces down on almost any crumbs of bread or meat thrown to him; but he prefers making little thievish raids on his own account on the loaf, cake, or cold joint which may be on the table. The thief is largely developed, apparently, in his nature. Whether he gets for himself, or is given a largish piece, he always hops off hurriedly with it, and pops down on the carpet with his quarry, for a private meal in a corner. He is very fond of cake-crumbs; and comes within an inch or two of one's hand to get what he has fixed his keen, suspicious, little eye on. And the most delightful part of his doings perhaps is, that when he has got a tid-bit or two he poses on the back of a vacant chair, or hops over the table-cloth or on the carpet, singing to us in a most sweet low tone—generally at the time when my canary up aloft is singing too. I never heard anything so soft and delightful as his little discoursing of song. It goes on quite continuously at times: and he varies the pitch according to his distance from us. Near, and on the table, all is of low key; but if he flies up to drink condensed vapour-drops from the topmost window-panes, he sings out clearly, or more so than one hears robins out of doors. We have tried to get him to drink from a wine-glass: but he is sadly puzzled by the transparent white glass in which the water is contained. He tries to get to it through the glass, in a very amusing if futile way. But if we place the glass on the carpet, and underneath the wires of a flower-basket, he at once sees the water, and drinks standing on the wire. Once or twice he has seen a brother robin fly near the window whilst he himself was within, and then he dashes fiercely towards the window, and utters a plaintive, long, low note as of defiance, and flies from window to window as though he was full of fight. What is that long, low note which I have mentioned? Have I given the true interpretation of it as a summons to battle? One day he uttered no other note than that: and we fancied he was ill and going to die; but latterly I have taken up the above idea concerning it, which I fancy is the truer interpretation. Our little friend has been coming and going for I should say six weeks; and it will cause sincere regret to our family circle if any cat should get into our secret, and break up our most pleasant intercourse with our little feathered friend.

(*To be continued.*)

